# Chats about Medical Museums

**Published:** 1887-05-07

**Authors:** 


### Chats about Medical Museums.

By One of the Crowd.

(Continued from p. 28, vol. ii.)

IV.—University College.

The medical museum at University College, Gower Street, is in some respects one of the finest in London. To the stranger who finds his way up into it, it seems a good way out of the world; and, as a matter of fact, few persons ever do find their way thither, unless they have some special motive for doing so. It is well worthy of a visit, however, by anyone having the smallest smattering of physiological knowledge, or any degree of interest in the many ills that flesh is heir to. Nor is it altogether wanting in points for those who would look round such a place—if there are any such—for the odd and curious alone. Near the door are some large glass cases, displaying a collection of what the uninitiated would probably take to be geological specimens. They are, of course, stones that have formed in the bodies of men or animals. Among these calculi is a large compact ball of hair, nearly as big as one's fist. This was taken from the body of a cow, and had probably been formed by the maternal licking of the calf. Such conglomerations are apparently not uncommon. There is a considerably larger one in the museum of the Royal College of Veterinary Surgeons in Kentish Town. But a far more remarkable accretion is a solid block of stone, shown on a table a short distance from these glass cases, and said to weigh originally no less than seven pounds and a half. This was taken from the body of a horse, which, in spite of his internal troubles, that occasionally took the form of what was supposed to be an attack of colic, lived for six-and-twenty years, the latter part of his time being spent in the service of a medical man. This formidable mass of stone has been cut through the centre, and the original cause of it was thus revealed. The unlucky horse had, it appears, somehow managed to get a nail into his internal economy, and round this calcareous matter had gradually accumulated in concentric rings, presenting, when cut through, very much the appearance of a section of the stem of a box-tree. Near this curious object is the heart of an elephant, hardened by a process of drying.

This museum consists of one large hall without galleries. It has a light and airy appearance, and its large floor-space is to some extent portioned off by movable screens ranged down its length. In the middle portion of the room thus shut in are a considerable number of very clever wax models, presenting all the chief forms of cutaneous disease—a frightful collection, as may well be imagined. But it is astonishing how soon one may come to regard the most repulsive objects in these places with the critical equanimity of the connoisseur, and to inspect them as works of art, rather than as manifestations of the most revolting phases of disease. These wax models are the work of Mr. Tuson, who spent a long time here depicting for the use of the students of the college typical cases met with in the hospital on the opposite side of Gower Street—the North London. The collection cannot rival that to be seen at Guy's, but it is a very valuable one nevertheless. This museum, too, has had the good fortune to be enriched by Sir Charles Bell's and Sir Robert Carswell's pathological drawings, as well as by Quain's preparations of the arteries. In anatomical dissections it claims to be unrivalled by any museum in London, excepting only that in Lincoln's Inn Fields. They are very numerous, and even to the untutored eye it is quite apparent that they are exceptionally good. As one mode of simplifying the study of the more intricate of the bones, they have adopted at this school of anatomy an idea that has not been met with in either of the other museums visited. They have the bones of the human body variously coloured. Each of the numerous sections comprising the skull, for instance, has a distinctive hue. One would think that Nature, so fond as she is of variety, might just as well have adopted this simple improvement herself. If every bone could be identified by its colour as well as its form, it would obviously greatly facilitate the work of the animal physiologist and comparative anatomist. The idea has, it appears, been imported from Paris, the chief market for "bones." There are immense difficulties in getting "subjects" in this country; in France they are not quite so squeamish, and there is a readier supply.

In this museum, as at St. Thomas's Hospital, they have portions of skeletons brought from the battle-fields of the Franco-German War, showing for the most part the effects of gun-shot in crushing and fracturing bones. Among other things they have here the skull of a German officer who went to war, but seems to have been dissatisfied with his chances of getting killed, and therefore blew his own brains out. There is the hole plainly seen in the roof of the skeleton's mouth and the top of the head. There also are bones that have been gnawed by a tiger in some far-away jungle, the remains of a feast off the body of some unlucky adventurer. Standing up in a glass case, on the other side of the room, is the skeleton of a once popular clown. Few things lend themselves to the imagination more readily than a skeleton, and one might easily persuade himself that the grim object is doing his best to look droll. Alas! alas! "Where be your gibes now? your gambols? your songs? Your flashes of merriment that were wont to set the table in a roar? Not one to mock your own grinning? Quite chopfallen?" Quite. Yet the rogue looks as though he wouldn't be if he could possibly help it. The great peculiarity of this man's skeleton is that he has hardly any thigh-bones to speak of. He has a headpiece which, if there is any truth in phrenology, is that of a clever man, as no doubt a popular clown must generally be. All his cleverness and his popularity, however, were not, it is to be feared, sufficient to prevent him dying friendless in some workhouse, or probably he would not have been grinning in this glass case. A decidedly interesting feature in this museum is the obstetric department of it. They have here an exceptionally numerous collection of abnormal forms of infant life; but perhaps on this subject enough was said in a previous article.

*(To be continued.)*

